# Supplementary material for: Long-term changes in body composition and their relationships with cardiometabolic risk factors: A population-based cohort study
Source: PLoS One. 2021 May 13;16(5):e0251486. doi: 10.1371/journal.pone.0251486 (PMC8118322; doi:10.1371/journal.pone.0251486)
Supplement: Attachment — Submitted filename: Response to the Reviewers.docx [file pone.0251486.s001.docx]

**Response to the Reviewers’ Comments**

PONE-D-20-38516

Long-term Changes in Body Composition and their Relationships with Cardiometabolic Risk Factors: A Population-Based Cohort Study

（Please note that the title has been changed from Body Composition Trajectory and Association with Cardiometabolic Risk Factors: a Population-Based Cohort Study to the above one）

**We are grateful for the thoughtful reviews provided by the external referees and valuable comments from the editors. We have carefully addressed each recommendation, and the manuscript is considerably stronger as a result of the modifications outlined below.**

The modified text is highlighted in tracked copy in the revised manuscript.

Requests from the reviewers:

Reviewer #1: Many thanks for the opportunity to review this manuscript. The comments below are intended as a constructive direction on improving the manuscript.

1. **Q: The manuscript will benefit from editing to tidy up grammar/spelling.**

**A:** Revised as suggested

We get help from Mark Cleasby, PhD, a native English speaker, from Edanz Group (https://en-author-services.edanzgroup.com/) for editing a draft of this manuscript .

Reviewer #2: Overview

The authors present an interesting analysis which considers different patterns of change in fat to fat-free mass ratio (trajectories) across 16 years in a population-based cohort in China. They relate the different trajectories to cardiometabolic outcomes. I think the concept and the results are interesting however I have some major concerns about the discussion and some of the conclusions which have been drawn from the results.

1. **Q: Furthermore, it is sometimes difficult to follow the points being made (particularly in the introduction & discussion). It is essential that the whole manuscript undergoes a thorough proof reading to ensure that all statements are written correctly in English and make sense as they are written.**

**A:** Revised as suggested

We get help from Mark Cleasby, PhD, a native English speaker, from Edanz Group (https://en-author-services.edanzgroup.com/) for editing a draft of this manuscript .

2. **Q: Introduction**

**Where the authors refer to ‘cardiovascular risk factors’, this includes dyslipidemia, diabetes, cholesterol and triglyceride measures; I would consider these as ‘cardiometabolic’ outcomes and suggest changing the general terminology throughout the manuscript.**

**A:** Revised as suggested

3. **Q: It would be good to define the ‘trajectory effect’ more clearly and mention potential biological underpinning for the trajectory effect itself, not just the negative consequences of obesity.**

**A:** Revised as suggested (Please see page 3 and 4)

4. **Q: F2MR, consider changing this to F2FFMR**

**A:** Revised as suggested

5. **Q: Methods**

**The first paragraph states that the first wave was 1989, then the ‘study population’ paragraph says baseline was 1993- this is a bit confusing, when was baseline?**

**A:** Revised as suggested (Please see page 4)

6. **Q: 3013 were included- how many were originally seen? Or, put another way, how many were excluded? In the flow chart it looks like 2792 participants were seen in 1993, I’m not sure I understand how 3013 participants can be included in this analysis?**

**A:** In this study, we included the participants who had at least two rounds of data. So the data was a maximum set of all 6 rounds.

7. **Q: Please insert the reference for ‘guidelines for the prevention and treatment of dyslipidemia in Chinese adults.**

**A:** Revised as suggested (Please see page 6)

8. **Q: Was there any distinction between type-2 and type-1 diabetes?**

**A:** In this study, we could not identify type-2 or type-1 diabetes accurately, but this study was a population-based study and type-1 diabetes usually was found in children and adolescents. Our study population was mainly in adults.

9. **Q: Note to editor: The group‐based trajectory modelling method was applied to the longitudinal body fat data using maximum likelihood method for model fitting and parameter estimation. I believe these are sound methods, however, I am not familiar with using them in my own work and therefore cannot fully confirm if this is appropriate.**

**A:** The group‐based trajectory modelling method was usually used for trajectory of longitudinal quantitative data, some references were quoted in introduction of our article.

10. **Q: The following sentence is unclear: ‘According to the trends of each trajectory group, low stability, high stability, increase and decrease, and increasing were designated in men. In women, low stability, high stability and increasing groups were designated (Figure 2).’In the text it could be clearer what each group means, i.e. ‘high stability’ means a high Fat to fat-free mass ratio without much variation between 1993 and 2009.**

**A:** Revised as suggested (Please see page 7)

11. **Q: Please clearly describe why fat to fat-free mass ratio was used and not just the fat % value?**

**A:** Revised as suggested (Please see page 4).

12. **Q: Results**

**I’m concerned about the high number of individuals who fall into the ‘high stability’ group. I am not a statistician so I cannot say if this invalidates the statistical methods in any way. It would be worth having the analysis verified by a statistician.**

**A:** We have asked a statistician for verification.

In table 4 and 5, the ORs of high stability are not very big and their CIs are narrow

And an article published in ‘Journal of clinical hypertension’ used the same method and its four groups number are 1045, 327,75 and 37, which is less than this study. Its sensitivity analysis was through Logistic regression.

Fan et, al. J Clin Hypertens. 2020;00:1–6. DOI: 10.1111/jch.14001

13. **Q: What is the justification for the adjustment strategy in tables 4 and 5? Why not have an age adjusted model before adjustment for other confounders (education, marital status etc..)?**

**A:** Revised as suggested (Please see page 4).

14. **Q: For a better understanding of mediation by age or original weight status, these factors should be sequentially adjusted for in separate models.**

**A:** Revised as suggested (Please see page 4).

15. **Q: In the conclusion the authors state ‘Our results also suggest that the impact of the adiposity trajectory on cardiometabolic profile is mediated by concurrent weight status.’ Is this a reference to tables 4 & 5 model 3? Could this association not also be mediated by age as age is included in model 3 along with weight status?**

**A:** Revised as suggested, we have separated age and weight status in model 2 and model 3.

16. **Q: Figures 1 & 2: I can’t see the figure legends anywhere in the submitted manuscript? These should be provided.**

**A:** Revised as suggested

17. **Q: Tables 2 & 3: the formatting is not quite right and makes the heading difficult to read.**

**A:** Revised as suggested

18. **Q: Discussion**

**The discussion is not well written and as such it is quite hard to follow the discussion points. The findings are discussed fairly superficially, more insight into potential physiological underpinnings for the different F2MR trajectories is necessary. For example, why might the high-stability trajectory group be associated with hypertension in men, independent of concurrent age and weight status? There is repetition of statements throughout the discussion which is unnecessary.**

**A:** Revised as suggested

19. **Q: Consider opening the discussion with a summary paragraph of the key results and why they are important/novel.**

**A:** Revised as suggested

20. **Q: Paragraph 2: ‘dyslipidaemia was mainly affected by concurrent weight status’ I don’t think you can draw this conclusion without separating age/weight adjustment in model 3.**

**A:** Revised as suggested (please see page 11)

21. **Q: The limitation section should also include the high number of participants who fall into the low stability group and low numbers in the other groups and discussion of potential ethnic differences in body composition trajectories.**

**A:** Revised as suggested (Please see Page)
