# Supplementary material for: Long-term changes in body composition and their relationships with cardiometabolic risk factors: A population-based cohort study
Source: PLoS One. 2021 May 13;16(5):e0251486. doi: 10.1371/journal.pone.0251486 (PMC8118322; doi:10.1371/journal.pone.0251486)
Supplement: Attachment — Submitted filename: Response to the reviewer.docx [file pone.0251486.s002.docx]

Response to the Reviewer’s Comments

Dear editor and reviewer,

We are grateful for the thoughtful reviews provided by the external referees and valuable comments from the editors. We have carefully addressed each recommendation, and the manuscript is considerably stronger as a result of the modifications outlined below.

The modified text is highlighted in tracked copy in the revised manuscript.

**Question 1:**

Introduction:
Line 45/46: “Millions of deaths can be ascribed to obesity worldwide [3], because it is associated with the development of cardiometabolic risk factors, such as dyslipidemia, diabetes, and hypertension[4, 5].”
This is not quite accurate. Dyslipidaemia, T2DM and hypertension are states of cardiometabolic disruption which increase the risk of adverse cardiovascular events.

**Answer 1：**Thank you for the question. we have revised as suggested. Please see line 45, page 3.

**Question 2:**

Line 46/47: “Fat mass is used to evaluate obesity[6]”
In ref #6 they defined obesity using a BMI cut-off, so I don’t think this statement makes sense.

**Answer 2：**Thank you for the question. we have revised as suggested. Please see line 47, page 3.

**Question 3:**

Line 61/62: “To date, few studies have characterized the trajectories of body composition and their associations with the development of cardiometabolic risk factors.”
I would say quite a large number of studies have tracked changes in body composition in longitudinal studies, however, I am unsure if this has previously been done in a Chinese population? Or if it has been done using the estimation of F2FFMR highlighting the importance of this work. Any more specific previous work on this should be referenced.

**Answer 3：**Thank you for the question. we have revised as suggested.
Please see line 62 to 66, page 3 to 4.

**Question 4:**
Methods:
(Q5&6) It is still not clear to me, from the written description and from Figure 1, how the authors arrived at the final sample size. What are the numbers in the box at the top right corner of fig 1?

**Answer 4：**Thank you for the question, we have revised as suggested. Please see figure 1.

We have revised figure 1 and make it clearer. In this study, we included 6 waves from 1993 to 2009 of the cohort. Participants we chose based on the wave 6 in 2009 which had blood biochemical results. There were 104, 63, 2, 62 new participants entered into the cohort in 1997, 2000, 2004 and 2006, respectively. The final sample size was 3013, which comprised of 2782 entered in 1993 and 231 new entered in 1997 to 2006.

**Question 5:**
“Those for whom data from at least two rounds of the survey were available were included.” How can you have a trajectory if you only have 2 measurements and such a wide age-range in this study sample? this should be explained, a supplementary information file might be helpful if the description is too lengthy for the main manuscript.

**Answer 5：**Thank you for the question.

The formation of trajectory trend was based on all the data involved in the analysis. The procedure of model fitting was as: Firstly, when all the data was imported in the Mplus, linear models and quadratic models were fitted. It showed that quadratic models had much smaller Bayesian information criteria (BIC) values across all models. Therefore, further analyses were based on the quadratic curve assumption and 4 classes in men, 3 classes in women. After the models and classes were determined, everyone was classified into a trajectory class.

To maximize the use of the data, we include 2 measurements in this study as other researchers did in theirs studies. In this study, 2 measurements of F2FFMR was only 4.8% (145/3013). After we exclude the data of 2 measurements, there was no obvious change in Figure 2.

1. Body mass index trajectories during the first year of life and their determining factors. *American Journal of Human Biology.* DOI: 10.1002/ajhb.23188
2. Body mass index trajectories during infancy and pediatric obesity at six years*. Annals of Epidemiology.*10.1016/j.annepidem.2017.10.008

**Question 6:**
When were the cardiometabolic outcomes measured (at which visit)?
Was waist-hip ratio measured in this study?

**Answer 6：**Thank you for the question, we have revised as suggested. The cardiometabolic outcomes were measured in wave 6 in 2009.There was no hip measure in this study. Please see page 6.

**Question 7:**
(Q8) This is not true. Type 1 Diabetes develops in childhood but is still present into adulthood.

**Answer 7：**Thank you for the information. I did some literatures review and found that China is one of the countries with the lowest incidence of type 1 diabetes. Although type 1 diabetes tends to develop in children, most of the new cases are diagnosed in adults.

*Weng J, Zhou Z, Guo L, Zhu D, Ji L, Luo X, Mu Y, Jia W; T1D China Study Group. Incidence of type 1 diabetes in China, 2010-13: population based study. BMJ. 2018 Jan 3;360:j5295. doi: 10.1136/bmj.j5295.*

In this study, the data was unavailable, we could not distinguish between type 1 diabetes and type 2 diabetes. There was no population-based data about the prevalence of type 1 diabetes in China, and it was 0.5% in US. So, the majority of diabetes in our study were T2DM. There might be a slight impact on our results.

*Xu G, Liu B, Sun Y, Du Y, Snetselaar LG, Hu FB, Bao W. Prevalence of diagnosed type 1 and type 2 diabetes among US adults in 2016 and 2017: population based study.BMJ.2018,362:k1497.doi: 10.1136/bmj.k1497.*

**Question 8:**
Results:
Table 5, statistically significant ORs not highlighted bold, as was done in Table 4.

**Answer 8：**Thank you for the question, we have revised as suggested. Please see page 14 and 15.

**Question 9:**

Discussion:
Lines 247-250: “The effect of F2FFMR on cardiometabolic risk is likely to accumulate slowly, and high F2FFMR in early life might have an impact on cardiometabolic health in later life.”
It is not clear if these statements are based on evidence presented in previously published work? Or are speculation.
**Answer 9:** Thank you for the question, we have revised as suggested. Please line 252 to 255, page 16.

**Question 10:**
Line 256/256: “Study of the F2FFMR indicates that the adverse effects of high fat mass can be offset by the protective effects of high fat-free mass.”
Line 267/268: “The present findings also suggest that the impact of F2FFMR trajectory on cardiometabolic outcomes is mediated by changes in body mass.”
Is it ‘changes in body mass’ or just ‘current weight’? I think you should revise your interpretation of your results.
**Answer 10:** Thank you for the question, we have revised as suggested. It should be current body mass.

**Question 11:**
Line 270: do you mean the ‘high stability’ group or do you mean ‘highly stable’?
**Answer 11:** Thank you for the question, we have revised as suggested. Please see page 17.

**Question 12:**

Line 294: I think the study sample from a Chinese only population living in China could be considered a strength of your study, given the previously published data from other parts of the world. There are other strengths that merit discussion as well.
**Answer 12:** Thank you for your advice. We added some points in it.

**Question 13:**
Line299/300: “Our results also suggest that the association between F2FFMR trajectory and the cardiometabolic profile is mediated by body mass.”
If the associations are all mediated by current body mass status, then why do we need to know about the trajectories? It looks like your data do not fully support this statement: in women (table 5), dyslipidemia/diabetes/hypertension are all still higher in the ‘High Stability’ group despite adjustment for weight in 2009?

**Answer 13:** Thank you for the question, we have revised as suggested. Please see page 18.
